# Burden of disease caused by local transport in Warsaw, Poland

**DOI:** 10.1016/j.jth.2015.06.005

**Published:** 2015-09

**Authors:** Marko Tainio

**Affiliations:** a UKCRC Centre for Diet and Activity Research (CEDAR), MRC Epidemiology Unit, University of Cambridge School of Clinical Medicine, Box 285 Institute of Metabolic Science, Cambridge Biomedical Campus, Cambridge CB2 0QQ, United Kingdom; b Systems Research Institute, Polish Academy of Sciences, Newelska 6, 01-447 Warsaw, Poland

**Keywords:** DALY, Transport, Air pollution, Injury, Physical activity, Noise

## Abstract

Transport is a major source of air pollution, noise, injuries and physical activity in the urban environment. The quantification of the health risks and benefits arising from these factors would provide useful information for the planning of cost-effective mitigation actions. In this study we quantified the burden of disease caused by local transport in the city of Warsaw, Poland. The disability-adjusted life-years (DALYs) were estimated for transport related air pollution (particulate matter (PM), nitrogen oxides (NO_*x*_), sulfur dioxide (SO_2_), benzo[a]pyrene (BaP), cadmium, lead and nickel), noise, injuries and physical activity. Exposure to these factors was based on local and international data, and the exposure-response functions (ERFs) were based on published reviews and recommendations. The uncertainties were quantified and propagated with the Monte Carlo method. Local transport generated air pollution, noise and injuries were estimated to cause approximately 58,000 DALYs in the study area. From this burden 44% was due to air pollution and 46% due to noise. Transport related physical activity was estimated to cause a health benefit of 17,000 DALYs. Main quantified uncertainties were related to disability weight for the annoyance (due to noise) and to the ERFs for fine particulate matter (PM_2.5_) air pollution and walking. The results indicate that the health burden of transport could be mitigated by reducing motorized transport, which causes air pollution and noise, and by encouraging walking and cycling in the study area.

## Highlights

• The health burden due to transport was quantified for the city of Warsaw, Poland.
• Burden was modeled through air pollution, noise, injuries and physical activity.
• Air pollution, noise and injuries caused a health risk of 58,000 DALYs per year.
• Physical activity was causing a health benefit of 17,000 DALYs per year.
• Health risks due to transport are larger than health benefits in urban environment.

## 1 Introduction

Transport is a major source of air pollution, noise, injuries, physical activity and other factors which have a direct impact on population health. In the Global Burden of Disease Study (GBD) 2010 fine particulate matter (PM_2.5_) air pollution, physical inactivity and lead (Pb) were the ninth, 10th and 25th most important risk factors, respectively (Lim et al., 2012). Road injuries were the 10th most important cause of death in the same study (Vos et al., 2012). In the further analyses of the GBD 2010 data, the Global Safety facility group concluded that motorized road transport deaths exceed those from the diseases such as HIV, tuberculosis or malaria, in the global level (Bhalla et al., 2014). In the “*European Perspectives on Environmental Burden of Disease*” (EBODE) study it was calculated that 80% of the environmental health burden in six European countries was due to PM_2.5_, noise, lead and benzene (Hänninen and Knol, 2011; Hanninen et al., 2014). Transport is an important source of all these factors.

The connection between transport and health through different factors has been recognized in several reviews, reports and commentaries (De Nazelle et al., 2011; Dora, 1999; Hyder et al., 2006; Kjellstrom et al., 2003; McCarthy et al., 2010; Thomson et al., 2008). For example, Dora (1999) commented that transport policies have important health consequences through air pollution, noise, injuries, climate change and by providing a safe environment for physical activity. The review of De Nazelle et al., (2011) built a conceptual model between active travel policies and health, and recognized that these are connected by air pollution, greenhouse gases, noise, heat, ultraviolet (UV) radiation, traffic hazards, physical activity, and other mediators.

Although the linkages between transport and health through different factors have been recognized, relatively few studies have quantified this relationship by taking into account several factors. For example, the aforementioned Global Safety facility group quantified the health effects of transport through injuries and PM_2.5_ air pollution alone (Bhalla et al., 2014). Some active transport studies have combined the health effects of air pollution, injuries and physical activity in urban and national levels (Macmillan et al., 2014; Rojas-Rueda et al., 2011; Woodcock et al., 2014, 2013, 2009). The most comprehensive health assessment that we are aware of is the Swedish Health Impact Assessment (HIA) of road transport that estimated the health effects of transport-related physical activity, air pollution, injuries, noise and climate change (Kjellstrom et al., 2008). Also some economic analyses, such as Cravioto et al., (2013) study from Mexico, have involved several factors in one assessment.

Most of the papers mentioned in the previous paragraph have assessed the health burden due to transport in national or international level. Many of the transport related factors, such as noise, injuries and physical activity, are local by nature, meaning that the health effects are felt near the source. Also, a significant part of the exposure to air pollution can occur near the roads in urban environments (Greco et al., 2007; Tainio et al., 2014). This indicates that local decision making has the potential to mitigate the adverse health effects caused by these factors; possibly more than national or international mitigation actions. To engage in such mitigation activities local decision makers require data on the magnitude of the health effects caused by transport, along with the relative importance of different factors to plan and prioritize the mitigation actions.

In this study the health effects of local transport for the local population are estimated for the city of Warsaw, Poland. The specific aims are: (i) to assess disability-adjusted life-years (DALY) caused by transport related air pollution, noise, injury and physical activity, and (ii) to assess DALYs for different air pollutants (particulate matter (PM), nitrogen oxides (NO_*x*_), sulfur dioxide (SO_2_), benzo[a]pyrene (BaP), cadmium, lead and nickel). The uncertainties of the main input variables are defined and the impact of that uncertainty to the model result is tested with sensitivity analyses.

## 2 Methods

### 2.1 Overview and the study area

The burden of disease due to local transport was estimated by modeling the exposure to transport related air pollution, noise, injuries and physical activity, and by calculating the changes in the background burden with and without these factors. Thus, the business as usual scenario was compared to the counterfactual scenario where the exposure to the transport fraction of these factors is zero. Air pollution, noise and injuries were estimated to cause negative health effects, expressed as DALYs. Physical activity due to active transport (walking and cycling) was estimated to improve health, represented as negative DALYs, i.e., reduction in disease burden. The main data sources are listed in Table S1 (Supplementary material).

The study area consisted of the administrative area of the city of Warsaw, Poland. In 2010, Warsaw had approximately 1.7 million inhabitants with an average population density of 3287 inhabitants/km^2^ (Statistical Office in Warsaw, 2012). Inside the city border approximately 2.6 million trips are made every 24-h period (Capital City of Warsaw, 2010). Of all these trips, 22% are made by walking, 23% by car, 55% by public transport, and 1% by other modes of transport (Capital City of Warsaw, 2010). According to TomTom (2012) European Congestion index Warsaw was the most congested city in the Europe.

### 2.2 DALY and the background burden data

In our study the health effects of transport are illustrated using DALYs. The DALY method has been developed in the Global Burden of Disease studies (Harvard School of Public Health et al., 1996; Murray and Lopez, 1997; Murray, 1994) and it has been used in several studies to combine and illustrate the health effects (Hanninen et al., 2014; Kjellstrom et al., 2008; Woodcock et al., 2014, 2013, 2009). DALYs have two components: years of life lost due to premature mortality or fatality (YLLs) and years lived disabled or injured (YLDs). The YLLs are calculated by comparing the age of the deceased person to the estimated life expectancy expected in a person of the same age and gender. YLDs are calculated by multiplying the number of diseases or injuries with the disability weight (DW) of that disease or injury and the duration (*D*) of the disease or injury. With lifelong diseases and injuries the duration used is the remaining life expectancy of the person.

The accurate calculation of DALYs depends on the availability of data, knowledge of the relationships between the exposure and the health outcomes, and other variables. In this study methods used in previous burden of disease studies (Hänninen and Knol, 2011; Hanninen et al., 2014), or recommended in the World Health Organization (2011) guidelines (Fewtrell et al., 2003), were followed when feasible, given the exposure data. See details for each factor in below chapters and overview in Table 1.

The data on the background level of different diseases and age group structure was estimated for this area from the Global Burden of Disease Study 2010 (2013) country files. GBD 2010 country files have information at the national level on background DALYs, YLLs, YLDs and deaths divided between different causes, gender and age groups. The background burden estimates are undiscounted and unweighted for age, following the Global Burden of Disease Study 2010 (2013) approach (Murray et al., 2012). The background burden for Warsaw was estimated from national data by taking account population size, gender and age structure differences between Poland and Warsaw. The population data was obtained from the Central Statistical Office (2012), Poland. The background burden data used in this study is presented in Table 2.

### 2.3 Air pollution

The exposure to air pollution generated by local transport was estimated for eight air pollutants: PM_2.5_ (fine particulate matter, particulate matter with aerodynamic diameter less than 2.5 µm), PM_2.5–10_ (coarse particulate matter, particulate matter with aerodynamic diameter in between 2.5 and 10 µm), nitrogen oxides (NO_*x*_), sulfur dioxide (SO_2_), benzo [a] pyrene (BaP), nickel (Ni), cadmium (Cd), and lead (Pb). These pollutants were chosen because of their potential to generate adverse health effects (Hanninen et al., 2014; Lim et al., 2012; United States Environmental Protection Agency, n.d.).

The exposure was estimated with the Gaussian puff dispersion model CALPUFF, version 5 (http://www.src.com/calpuff/calpuff1.htm). Details of the dispersion modeling and exposure assessment has been described in Holnicki and Nahorski (2013) and Tainio et al. (2014), respectively. In short, the dispersion modeling was done with 1×1 km^2^ spatial resolution over the study area. The emission data for different air pollutants was obtained from EKOMETRIA, Poland (http://www.ekometria.com.pl/) and it included both tailpipe emissions of pollutants as well as PM raised by road traffic. The population data was obtained from the European Environment Agency (2008). The exposure was calculated as the population-weighted mean concentration of pollutants in the air. The annual average exposure for these pollutants is presented in Table S3 (Supplementary material).

For the health calculations the PM air pollution was divided to two fractions, PM_2.5_ and PM_2.5–10_, because these fractions cause different health effects (see details below). PM_2.5_ fraction includes primary PM_2.5_ emitted from car exhausts, secondary PM formed from the gaseous SO_2_ and NO_x_ emissions, and the fine dust raised from the road due to transport. PM_2.5–10_ contains primary PM emitted from car exhausts and fine dust raised by the traffic. For other air pollutants only exhaust emissions were taken into account.

#### 2.3.1 Health calculations for air pollution

The exposure-response functions (ERFs) for different air pollutants and health outcomes are summarized in Table 1 and the background burden data in Table 2. We provide an overview of the methods and data sources below. Further details are provided in the Supplementary material (Chapter 2).

For the PM air pollution we estimated increased natural-cause mortality due to PM_2.5_, new cases of chronic bronchitis due to PM_2.5_ and PM_2.5–10_, restricted activity days (RADs) due to PM_2.5_, and lower respiratory symptom (LRS) days for the school children and for the adults due to PM_2.5_ and PM_2.5–10_. The same health outcomes were modeled in the EBoDE study (Hanninen et al., 2014).

The ERF for natural-cause mortality was based on the multicenter European Study of Cohorts for Air Pollution Effects study (Beelen et al., 2013; Table 1). Beelen et al. (2013) combined 19 cohort studies in Europe and adjusted hazard rates for gender, calendar time, smoking status, smoking intensity, smoking duration, environmental tobacco smoke, fruit intake, vegetable intake, alcohol consumption, body-mass index (BMI), educational level, occupational class, employment status, marital status and area-level socioeconomic status. Most of the cohort studies included in the Beelen et al. (2013) were for adult populations and therefore we predicted an increase in natural-cause mortality for adults aged 30 years and over.

For the other health outcomes caused by PM exposure we adopted unit risk (URs) values from the Clean Air for Europe (CAFE) program report (Hurley et al., 2005). The URs are summarized in Table 1 and the DWs and Ds in the Table S2 (Supplementary material). To calculate lower respiratory symptom (LRS) days for adults we assumed that 30% of adult population have chronic respiratory symptoms, with uncertainty range from 20% to 50% (Hurley et al., 2005).

The adverse health effects of NO_*x*_ were estimated with the same method as the natural-cause mortality for PM_2.5_. The ERF for NO_*x*_ was adopted from European Study of Cohorts for Air Pollution Effects (Beelen et al., 2013; Table 1).

For SO_2_, BaP, Cd and Ni we predicted an increase in lung cancer cases based on the relative risk or unit risk approach. For SO_2_ we adopted relative risks from the Nafstad et al. (2003) study that followed a cohort of 16,209 Norwegian men with 27 years of follow up time. For BaP we adopted mean unit risk values from the World Health Organization (2000) Air Quality guidelines for Europe and for Cd and Ni from the ExternE year 2005 update (Bickel and Friedrich, 2005). For uncertainties see Table 1 and the Supplementary material. For the BaP, Cd and Ni new cancer cases per year were converted to DALYs by multiplying the number of new cases per year with the mean DALY loss of one lung cancer (21.4 DALYs per lung cancer death), estimated from the Global Burden of Disease Study 2010 (2013) data for Poland.

The adverse health effects of Pb exposure were estimated by following the WHO burden of disease guidelines for Pb (Fewtrell et al., 2003). In the guideline, exposure to Pb was associated with the mild mental retardation (MMR) for children and increased cardiovascular diseases for adults. Due to the non-linear dose–response relationship between Pb and the associated health effects, we first calculated total health burden due to Pb in the study area and then estimated fraction of the burden caused by local traffic related Pb emissions. Details are provided in the Supplementary material.

The background blood level Pb concentration in children was estimated from Barton (2011). In that study the Pb and Cd levels in blood, hair and teeth were measured from 300 preschool age children in Southern Poland (Table S4, Supplementary material). For background blood level concentrations for adults, the regional blood level of 9.2 µg/dl was used with the standard deviation of 3 for adults in Poland, Turkey and Yugoslavia, as presented in the appendix of the Fewtrell et al. (2003) (Table S4, Supplementary material).

The average Pb concentrations in the air were converted to blood Pb levels by assuming that 1.0 µg/m^3^ increase of Pb in the air leads to 50 μg/L increase in blood level Pb levels, following a similar approach to that taken in ExternE (Bickel and Friedrich, 2005). For children, 1.4% of the total burden of Pb and for adults 6.1% of the total burden was estimated to be due to air pollution emissions from local transport.

### 2.4 Traffic injuries

The DALYs due to traffic injuries in Warsaw were estimated by first calculating the DALY given the traffic fatality rate and then by multiplying the number of fatalities by this rate. This approach allowed us to estimate the DALYs due to fatalities (YLLs) and injuries (YLDs) when only traffic fatality data was available.

The number of fatalities in Warsaw was approximated by combining data from local traffic reports (Capital City of Warsaw, 2010) and personal information from the police. By combining this information, the number of transport fatalities in the study area for year 2009 was estimated to be 63, 3, 25, 4 and 2 for pedestrians, cyclists, car occupants, public transport users and for the users of other modes, respectively. The DALY to fatality rate was calculated from the Global Burden of Disease Study 2010 (2013) country data for Poland by dividing the number of DALYs due to “Road injury” with the number of deaths due to “Road injury”. The average DALYs per fatality was estimated to be 65 DALYs.

### 2.5 Noise

The DALYs caused by noise were estimated by following the recommendations of the World Health Organization (2011) expert group for noise. The WHO group concluded that the noise is associated with increases in cardiovascular disease, cognitive impairment in children, sleep disturbance, tinnitus and annoyance. In this study, noise-related DALYs due to sleep disturbance, annoyance and cardiovascular diseases were estimated. These three diseases accounted approximately 97% of total environmental noise related DALYs in the Europe in the World Health Organization (2011) expert group calculation. Details of the exposure data and the methods for each health outcome are described below and in the Supplementary material.

The noise exposure data for Warsaw was obtained from the European environment information and observation network (Eionet) noise database (European Environment Agency (EEA), n.d.). Eionet collects and summarizes the noise data that European Union (EU) member states have to submit to the EU following the Environmental Noise Directive (European Directive 2002/49/EC). For each major EU city the database has information on the number of people exposed to different day, evening and night (*L*_den_) and night (*L*_night_) (23:00–7:00) noise levels. The noise levels are reported with the A-frequency weighing decibels (dB(A)). The *L*_den_ and *L*_night_ data for Warsaw is presented in Table S6 (Supplementary material). The source of noise is road transport, as defined in the Eionet (European Environment Agency (EEA), n.d.). Exposure to railway, airport and industry noise was not taken into account.

The DALYs due to noise related ischemic heart diseases were estimated based on the day, evening and night noise (*L*_den_) exposure levels. The odds ratios (ORs) were adopted from the Appendix 1 of the Burden of disease from environmental noise guide (World Health Organization, 2011). The ORs were based on polynomial fit of the association between road traffic noise and incidence of myocardial infarction (World Health Organization, 2011).

High sleep disturbance (HSD) due to road traffic noise was estimated by applying the average nighttime noise level to the polynomial function obtained from Miedema et al. (2003). Function was predicted based on self-reported sleep disturbance from 15 different data sets. Similarly, for annoyance the polynomial function, obtained from (Miedema and Oudshoorn, 2001), was used to estimate percentage of population annoyed in the study area (Table S7, Supplementry material). For annoyance the day, evening and night noise exposure levels (*L*_den_) were used. See Supplementary material for details.

### 2.6 Physical activity

The health benefits of physical activity were estimated based on the average active transport levels of the population. Active transport refers here to walking and cycling, including short walking trips to and from public transport. The level of physical activity was summarized with the metabolic equivalent of task (MET) measure. One MET is the ratio of work metabolic rate to a standard resting metabolic rate of 1.0 kcal/(kg×h)^−1^ (Ainsworth et al., 2011). Typical MET values vary in between 0.9 (sleeping) to 23 (fast running) (Ainsworth et al., 2011).

#### 2.6.1 Physical activity due to active transport

The year 2005 Warsaw traffic survey was used to estimate the number of cycling, walking and public transport trips in the study area (Capital City of Warsaw, 2005). The average number of trips per day per person was estimated by combining information from the number of households in the survey, the average number of people living in one household and the number of trips per household per day (Capital City of Warsaw, 2005). The results (1.9 trips per day for week day and 1.5 for Saturday) were used as median values to estimate the number of trips per day. To account for uncertainty minimum and maximum trips per day per person were estimated to be 1 and 4, respectively, for both week days and Saturday. As a result, the Warsaw population was estimated to have 27 million trips per week.

The METs due to active transport were estimated by multiplying the number of trips on different modes, average time per trip and METs generated while walking and cycling together. Table S8 (Supplementary material) summarizes the data used. The short walking trips associated with using public transport were taken into account by assuming that each public transport trip would include 1 to 10 min of walking, with a median time of 5 min per trip. A similar approach has been used before in Rojas-Rueda et al. (2011).

On average the study population gained 5.1 MET h/week by walking and 0.6 METh/week by cycling. From the walking generated METs, 1.7 MET h/week was due to walking trips to and from public transport.

#### 2.6.2 Exposure-response function for physical activity

The health benefits of physical activity were estimated based on the ERFs obtained from the review and meta-analysis of Kelly et al. (2014). Based on the meta-analysis of 14 studies for walking and 7 studies for cycling, Kelly et al. (2014) estimated that the reduction in the all-cause mortality due to 11.25 MET h per week increase in walking and cycling would be 11% and 10%, respectively. The ERFs in Kelly et al. (2014) were adjusted for other physical activities which allows calculation of health benefits without assessing the baseline physical activity levels of the population. The background disease burden data included YLLs from by all causes for both men and women, and all age groups (Table 2)

### 2.7 Sensitivity analysis

Calculations were implemented with the Monte Carlo simulation program Analytica (Lumina Decision Systems, Inc.), version 4.5. Uncertainty was propagated through the model with 50,000 iterations. Each uncertain input parameter has been described in the text and in the Supplementary material. The sensitivity of the model to each uncertain input variable was tested and illustrated with tornado plots by calculating the model results with the median, low and high estimate of that input variable.

## 3 Results

**Fig. 1:**
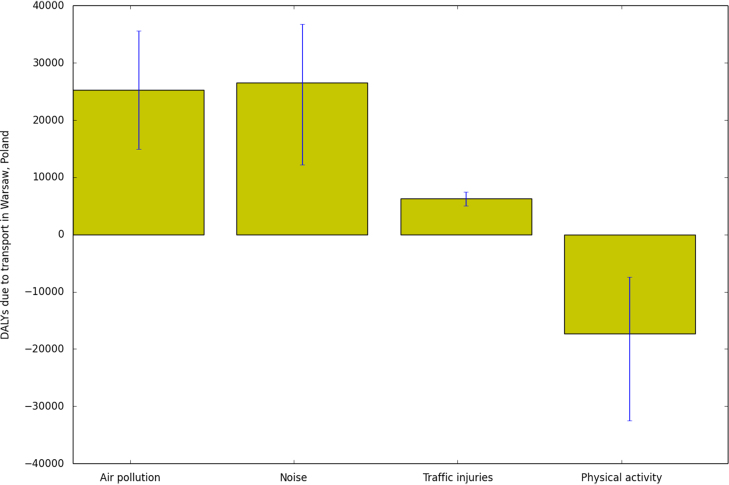
Total DALYs in study area due to four different factor groups. Negative DALYs mean health benefits.

Local transport was estimated to cause 41,000 DALYs (95% confidence interval from 19,000 to 62,000 DALYs) per year in Warsaw when health risks and benefits were combined (Table 3, Fig. 1). Air pollution, noise and traffic injuries were estimated to cause approximately 25,000; 26,000 and 6300 DALYs per year, respectively, and physical activity a health benefit of 17,000 DALYs per year.

The combined burden of air pollution was 25,000 DALYs (Table 3). From this 79% was due to PM air pollution (both PM_2.5_ and PM_2.5–10_ fractions), 20% due to NO_*x*_ and 0.8% due to Pb. Other air pollutants had insignificant impact to the burden. Approximately 67% of all the DALYs due to air pollution were attributable to natural mortality caused by PM_2.5_. Of the morbidity outcomes, chronic bronchitis was the most important one and accounted for nearly 9% of the total burden. About 87% of total burden was captured by estimating natural mortality loss caused by PM_2.5_ and NO_*x*_.

The health burden due to noise was caused by the annoyance (49%), sleep disturbance (38%) and ischemic heart diseases (13%). For the noise burden the uncertainties were large so that for the annoyance the mean DALY was 12,000 and the 95% CI ranged from 4000 to 27,000. This uncertainty was caused by relatively high uncertainty of disability weight for annoyance (Table S2, Supplementary material).

The traffic injuries and fatalities were estimated to cause 6300 DALYs per year in the study area. Of the total burden, 65% were suffered by pedestrians whereas 25% affected drivers and other occupants of private cars.

The physical activity due to active transport was estimated to cause a health benefit of 17,000 DALYs per year by reducing the all-cause mortality (Table 3). The health benefits of cycling and walking were 1800 DALYs and 15,000 DALYs per year, respectively, reflecting mode share differences (Table S8, Supplementary material). About one third of the benefits of walking were due to short walking trips to and from public transport.

**Fig. 2:**
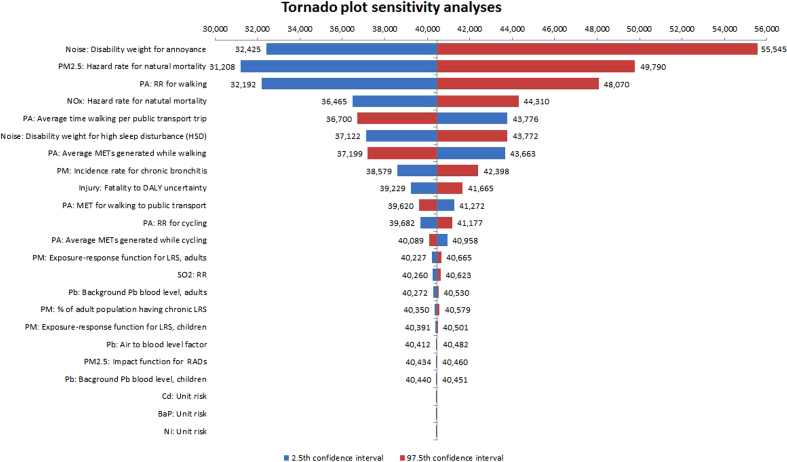
Tornado plot sensitivity analysis.

The input variable “Noise: Disability weight for annoyance” had the largest impact on the model result in the sensitivity analysis (Fig. 2). That input variable defines the DW for the annoyance and therefore modifies greatly the health burden caused by the noise. The two other significant input variables were related to ERFs for PM_2.5_ and walking (Fig. 2). Most of the other uncertainties had small or insignificant impact on the model results (Fig. 2).

## 4 Discussion

Local transport in Warsaw was estimated to cause approximately 41,000 DALYs a year for the population of the Warsaw. Taking into account only the health risks caused by air pollution, noise and traffic injuries, the total health burden would be 58,000 DALYs. This accounts for 11% of all the DALYs in the study area (Table 2). The main risks were PM_2.5_ air pollution and noise, indicating that the health of local population could potentially be improved best by reducing PM_2.5_ emissions and noise from motorized transport.

The health effects were calculated by combining the risks and benefits of active transport and motorized transport. The mode-specific health effects were not calculated due to a lack of data on the contribution of different travel modes to air pollution and noise. However, some general conclusions can be made by comparing the available information. For example, all the health benefits are related to active transport, especially to walking. Most of the risks are caused by the air pollution and noise generated by motorized transport. Thus, the health risks and the health benefits are caused by different travel modes. This indicates that the health risks caused by transport could be reduced if more trips included a form of active transport. Several previous studies have examined this issue in different contexts and come to a similar conclusion (Macmillan et al., 2014; Rojas-Rueda et al., 2011; Woodcock et al., 2014, 2013, 2009).

The health benefits of active transport were calculated separately for walking and cycling. Some previous studies, such as (Kjellstrom et al., 2008), have assessed the negative effect of transport related physical activity by accounting the fraction of motorized trips that could have been completed on foot or by bicycle, and then calculating the loss of health due to this missing physical activity.

Traffic fatalities and injuries were contributing less to the total burden than the three other factor groups (Fig. 1). Fatalities and injuries are an important and visible risk of transport and a fair amount of work has been done to raise the awareness of the health burden of injuries at the world (World Health Organization, 2004) and European level (Racioppi et al., 2004). The threat of injuries might also decrease peoples willingness to use active transport (Transport for London (TFL), 2012) and this way reduce the health benefits. Therefore our intention is not to say that injuries are not important but rather to say that in addition to the significant burden of fatalities and injuries, transport also causes other negative externalities such as air pollution and noise, and in some conditions, such as in the urban areas, these other externalities might create as large or even a larger burden than direct injuries.

The health effects of different factors were combined with the DALY method. This allowed various health effects, such as additional mortality, restricted activity days, annoyance and traffic fatalities, to be compared. In practice the comparison of diseases and injuries is done with disability weights that define the severity of disease and injury in comparison to being death. This might lead to some controversial results when comparing mild but common and severe but rare health effects. For example, in this study approximately 1100 people were estimated to die due to PM_2.5_ air pollution emitted from local transport and nearly 260,000 people were estimated to be annoyed due to noise; the resulting total DALYs are of a similar magnitude for both factors (Table 3).

### 4.1 Confounding and interaction between factors

A number of the factors combined in this study were related to the same diseases. For example, several pollutants were estimated to increase lung cancer incidence and mortality (see Table 1), and several air pollutants and noise were estimated to increase cardiovascular disease. This raises questions related to possible confounding and interaction of these factors. Confounding means that the health effect observed for one factor (e.g., noise) might be due to another factor (e.g., air pollution), if exposure for these factors is correlated. Interaction means that if two factors are having an impact to same health outcome, the combined effect might be higher than that of each effect individually. The possible impact of confounding and interaction on our results is discussed below.

DALYs due to air pollution were calculated combining the health effects of different air pollutants together. This approach has at least two challenges. First, the epidemiological studies examining the health effects of different air pollutants are sensitive to the correlations between pollutants. In this study the hazard rates for mortality due to PM_2.5_ and NO_*x*_ were both based on Beelen et al. (2013) (Table 1). In Beelen et al. (2013) the hazard rate for PM_2.5_ remained statistically significant when adjusted for PM_2.5–10_ and NO_2_, but the effect of NO_2_ was not statistically significant when adjusted to PM_2.5_ and PM_2.5–10_. This could indicate that the hazard rate estimated for NO_*x*_ in Beelen et al. (2013) could be due to confounding with PM_2.5_ rather than the independent effect of NO_*x*_. However, a recent meta-analysis examining the confounding between NO_2_ and PM showed small change in the effects of NO_2_ when PM exposure was taken into account (Faustini et al., 2014). For our study, the possible confounding between NO_*x*_ and PM exposure could have up to 20% impact to total health burden of air pollution and therefore this issue is of high importance for future assessment studies.

The second air pollution related challenge for similar studies is that it is unclear to what extent the health risks of BaP and metals (Cd, Ni and Pb) are included in the health risks of PM. PM air pollution is a mixture of solid and liquid particles with varying components, including BaP and metals, and due to this reason the hazard rate for PM_2.5_ might include the adverse health effects of these components. For our calculation this possible double counting would have only a small impact on the total burden because the combined effect of BaP and metals was less than 1% of the total burden.

For the noise most of the DALYs were due to annoyance and HSD, and it is likely that these two outcomes are related at the individual level. This might mean that by adding the health burden due to these two health outcomes together, the true burden was overestimated. In the burden of disease due to environmental noise study the total burden of noise in Europe was estimated to be in between 1.0–1.6 million DALYs, depending on how much the different health outcomes were estimated to overlap (World Health Organization, 2011). In the EBoDE study (Hänninen and Knol, 2011; Hanninen et al., 2014), the environmental noise was associated only with HSD and ischemic heart disease, without annoyance. If annoyance was left out from the calculation, or assumed that annoyance and HSD are perfectly correlated, the total burden due to road related noise would drop to 13,000–17,000 DALYs per year. Thus, noise burden would still be 2–3 times higher than the burden of traffic fatalities and injuries.

Air pollution and noise are both associated with cardiovascular health outcomes and in an urban environment noise and air pollution levels are moderately correlated (Allen et al., 2009). Tetreault et al. (2013) examined this by reviewing nine studies that had assessed confounding between air pollution and noise for cardiovascular diseases. They concluded that, based on what is currently known, the confounding effect is small (Tetreault et al., 2013). They also examined interactions between noise and air pollution to see if the combined effect of air pollution and noise might be greater than the two individual effects. Only two studies had examined interaction between noise and air pollution and both of the studies reported a non-significant interaction (Tetreault et al., 2013).

In conclusion, it is unclear to what extent possible confounding and interaction between factors have modified our results. Still, even by making highly conservative assumption that the health effects of NO_*x*_ are due to PM_2.5_ and that annoyance includes the health effects of HSD, the total burden of noise and air pollution would be 37,000 DALYs per year; a large enough burden to justify mitigation actions.

### 4.2 Comparison to previous studies

Few previous studies have examined the impact of transport on health through several factors. One of the most similar one to our analysis is the Kjellstrom et al. (2008) study that estimated the health effects of road transport in Sweden through traffic injuries, air pollution, noise, physical inactivity and climate change. Health risks due to physical inactivity were estimated by assuming the fraction of the private car trips that could be done with active transport and then estimating the loss of physical activity, and health, due to these car trips. The health effects of air pollution were based on the mortality risk due to PM_2.5_. Health risks of climate change were approximated from the WHO Global climate change risk estimates (McMichael et al., 2004).

The combined effect of injuries, air pollution and noise for the Swedish population was estimated to be around 100,000 DALYs per year, accounting of 6% of all DALYs (Kjellstrom et al., 2008). In our study same figure is 7.5%, if the health risks and benefits are combined. Results differ more substantially when factor-specific estimates are compared (Table S9, Supplementary material). DALYs per million inhabitants were similar between Kjellstrom et al. (2008) and our study in terms of injuries but for other factors the health effects were much higher in this study. Partly this could be explained by methodological differences. For example, in Kjellström et al. DALYs due to noise were calculated only through the increase of ischemic heart disease. Since annoyance and HSD generated large fraction of DALYs in the present study, our DALY estimates for noise are much larger per million inhabitants.

However, methodological differences cannot explain all the differences between studies. Even if only the ischemic heart disease risks for noise were taken into account, the DALYs per million inhabitants would be 2000 while in Kjellström et al. the corresponding number is 450. The difference is also too large to be explained by differences in the background burden, traffic or cultural differences between Warsaw and Sweden. This difference is probably, at least to some extent, due to differences between national and urban estimates. While in Kjellström et al. DALYs due to road transport were estimated for the whole country, including urban and non-urban areas, this study focused on one urban area where air pollution and noise-associated health effects are estimated to be higher than the same effects at the national level.

Our result also differs from the Global burden of disease due to motorized road transport study (Bhalla et al., 2014). In that study DALYs due to traffic related PM_2.5_ air pollution and transport crashes were calculated at the global level. The effect of physical activity was discussed but unquantified. Transport crash estimates were based on Global Burden of Disease Study 2010 (2013) data and the exposure to traffic related PM_2.5_ was approximated from global PM_2.5_ concentrations used in the Global Burden of Disease Study 2010 (2013) (Bhalla et al., 2014). Globally, transport related deaths were dominated by crashes. In Western Europe 44% of all the deaths were due to PM_2.5_ and 56% due to fatalities. Bhalla et al. (2014) estimated that the exposure to PM_2.5_ was underestimated in their study resulting in underestimation of the health burden. This underestimation of the exposure of PM_2.5_, together with differences between national and urban levels, explains the differences between studies

Several previous studies have examined the health risks and benefits of active transport in national and urban context (Macmillan et al., 2014; Rojas-Rueda et al., 2011; Woodcock et al., 2014, 2013, 2009). In most of these studies researchers have examined the effect of an increase of active transport and they have found out that the health benefits of physical activity have been higher than the risks of crashes and air pollution. In the present study the benefits of physical activity are lower than the risks of air pollution, but the health benefits of the walking and cycling are generated by small fraction of population who walk and cycle in the study area (see Table S8, Supplementary material). This indicates that even small increase in walking and cycling would generate large health benefits, as has been noted in the aforementioned studies (Macmillan et al., 2014; Rojas-Rueda et al., 2011; Woodcock et al., 2014, 2013, 2009).

### 4.3 Uncertainties and further development

A number of approximations were required to quantify the risks and benefits in this study. The quantification of uncertainties and testing of the model using sensitivity analyses (Fig. 2) provided some information on the consequences of some of the assumptions but not all could be quantified or tested. Some of these limitations and a discussion of their implications for the results is provided below.

The highest uncertainties in the sensitivity analysis were related to the disability weight for noise and ERFs for PM_2.5_ and walking. From these three input parameters, DW for annoyance is of highest importance because of possible correlation between annoyance and HSD, as discussed earlier. Thus, it is uncertain if annoyance should be included in the calculation at all. This clearly creates uncertainty around the burden caused by noise and requires more epidemiological evidence on the associations between noise and different outcomes.

For other two input variables causalities are better established. Physical activity, for example generated by walking, is reducing the health burden, and exposure for PM_2.5_ air pollution is increasing the health burden. The exact magnitude of these effects, expressed as ERF uncertainty (Table 1), is uncertain and the results are sensitive to small changes in ERFs. Still, even with extreme values natural-cause mortality due to PM_2.5_ was estimated to cause nearly 8000 DALYs and walking generated physical activity a health benefit of 6000 DALYs. Thus, the health risk and benefits would be smaller but still significant when compared e.g., to health burden of traffic injuries (6300 DALYs, Table 3).

For a number of parameters in this model some indicative data from other areas or setting were used. For the blood level Pb concentration, data was based on the measurement done in southern Poland for children (Barton, 2011) and on the regional blood level obtained from the WHO guideline for adults (Fewtrell et al., 2003). Neither of these are specifically for the Warsaw population for the period from which the background health data were drawn (Table S1, Supporting material). However, because Pb had only a minor impact on the model results, the uncertainties in the blood levels had only a small impact on the model results (Fig. 2).

The inclusion and exclusion of certain factors in the model also leads to uncertainty. For example, from air pollutants carbon monoxide (CO) and ozone (O_3_) could have been included since both of these pollutants have been associated with adverse health effects (World Health Organization, 2006, 2000). Unfortunately no information on the increase in concentration due to transport-related CO and O_3_ for the study area was available. Our assumption is that even if CO and O_3_ were included, the health burden due to air pollution would have been dominated by PM air pollution and the total burden would have been similar in magnitude.

However, the use of simplified methods and data from outside the study area means that these results are more likely to be reproducible in other locations. The background burden data is available for all the countries in the world, all the exposure-response functions are transferable to other study locations, and exposure data for noise is available in similar format for all the large EU cities. Similarly, traffic fatality data should be available for many locations and the urban air pollution measurement networks would allow for estimation of the health burden due to transport-related air pollution in several urban areas and nations in Europe and beyond. With the help of tools like the Health economic assessment tool (HEAT) for cycling and walking (Kahlmeier et al., 2014) the contribution of physical activity to the total burden could be included in these calculations without extensive activity surveys and methods. Regardless of all the uncertainties, such analyses provide valuable information for the planning of the more extensive studies and mitigation plans.

## 5 Conclusions

This study provides an insight into the magnitude of the disease burden caused by transport in an urban area through different factors. Based on the results of this study it can be concluded that in an urban environment, transport-generated air pollution, noise, injuries and physical activity are all have a significant health effect. Of all the different air pollutants most of the burden is due to PM_2.5_ air pollution. The health risks of air pollution and noise are mainly related to motorized transport whilst the health benefits of physical activity are related to walking and cycling. This suggests that the health burden of transport could be reduced by encouraging more people to use active transport instead of motorized transport.

## Figures and Tables

**Table 1:** Summary of exposure-response functions (ERFs) used in the study.

Factor	**Health endpoint**	**Age group**	**Type of ERF**	**ERF**	**Details and references**
PM_2.5_	Natural-cause mortality	30+	HR	1.07 (95% CI 1.02–1.13)	Change in hazard rate per 5 µg/m^3^ change in PM_2.5_ concentration. Adopted from Beelen et al., (2013); Table 4, model 3.
PM_2.5_, PM_2.5–10_	New cases of chronic bronchitis	30+	UR	5.33×10^−5^ (−0.17×10^−5^–11.3×10^−5^)	New cases of chronic bronchitis per year per persons per 1 µg/m^3^ change in PM_10_concentration. UR is adopted from the CAFE (Hurley et al., 2005), which calculated URs based on the Abbey et al. (1993). Uncertainty range from CAFE.
PM_2.5_	Restricted activity days (RADs)	15–64	UR	0.090 (0.079–1.013)	RADs per person per 1 µg/m^3^ change in PM_2.5_ concentration. Adopted from CAFE (Hurley et al., 2005) and based on OSTRO (1987). Uncertainty range from CAFE.
PM_2.5_, PM_2.5–10_	LRS days for school children	5–14	UR	0.186 (0.186–0.277)	Extra symptoms days per year per child aged 5–14, per 1 µg/m^3^ change in PM_10_. Adopted from CAFE (Hurley et al., 2005) and based on RR from Ward and Ayres (2004). Uncertainty range from CAFE.
PM_2.5_, PM_2.5–10_	LRS days for adults	15+	UR	0.13 (0.015–0.243)	Extra symptom days per year per adult with chronic respiratory symptoms per 1 µg/m^3^ change in PM_10_ concentration. Adopted from CAFE (Hurley et al., 2005) and based on the meta-analysis done in the CAFE project. Uncertainty range from CAFE.
NO_*x*_	Natural-cause mortality	30+	HR	1.02 (95% CI 1.00–1.04)	Change in hazard rate per 20 µg/m^3^ change in NO_x_ concentration. Adopted from Beelen et al. (2013); model 3 results for NO_*x*_ in Table 4.
SO_2_	Lung cancer	All	RR	1.01 (0.94–1.08)	RR for developing lung cancer per 10 µg/m^3^ increase in SO_2_ concentration. Based on Nafstad et al. (2003).
BaP	Lung cancer	All	UR	8.7×10^−5^ (1.0×10^−5^–10×10^−5^)	A life time risk of developing lung cancer per ng/m^3^ change in BaP concentration. Based on World Health Organization (2000) Air Quality guidelines for Europe. Uncertainty range from Bostrom et al. (2002).
Cd	Lung cancer	All	UR	1.8 ×10^−3^ (1.0×10^−3^–1.8×10−^3^)	A life time risk of developing lung cancer per µg/m^3^ change in Cd concentration. Based on ExternE (Bickel and Friedrich, 2005). Uncertainty based on the Takenaka et al., (1983) and author judgment.
Ni	Lung cancer	All	UR	2.4×10^−4^ (1.1×10^−5^–2.4×10−^4^)	A life time risk of developing lung cancer per µg/m^3^ change in Ni concentration. Based on United States Environmental Protection Agency, n.d. IRIS database. Uncertainty based on Peto et al. (1984) and Chovil et al. (1981).
Pb	Mild Mental Retardation (MMR)	0–1	Specific, see text for details.	Specific, see text for details.	Method from Fewtrell et al., (2003), dose–response data from Schwartz (1994).
Pb	Cardiovascular disease	15–79	RR	See Table S5 (Supplementary material).	Method from Fewtrell et al., (2003). Based on Pruss-Ustun et al., (2004).
Traffic injuries	Fatalities and injuries	All	Specific, see text for details.	Specific, see text for details.	-
Noise	Cardiovascular disease	All	OR	See Table S6 (Supplementary material) for details.	Data, method and RRs from Babisch, (2006) and World Health Organization (2011).
Noise	Sleep-disturbance	All	Specific, see text for details.	Specific, see text for details.	Method and ERF from World Health Organization (2011).
Noise	Highly annoyed	All	Specific, see text for details.	Specific, see text for details.	Method from World Health Organization (2011). Exposure-response function from Miedema and Oudshoorn (2001).
Physical activity	All-cause mortality	All	RR	0.90 (0.85–0.95) for walking and 0.90 (0.86–0.94) for cycling	Dose–response function from Kelly et al. (2014). RR׳s are for 11.25 METhs per week.

**Table 2:** Background disease burden data for the study area, based on the Global Burden of Disease Study 2010 (2013).

**Disease**	**Age group**	**Burden measure (DALY, YLL, YLD)**	**#**	**Factor**	**Details**
All causes	All	DALY	547272	-	Total (all causes).
All causes	All	YLL	315936	Physical activity	Total (all causes).
Natural-cause mortality	30+	YLL	265705	PM_2.5_, NO_x_	Communicable, maternal, neonatal, and nutritional disorders; non-communicable diseases
Lung cancer	All	DALY	22605	SO_2_	Trachea, bronchus, and lung cancer.
Ischemic heart disease	All	DALY	65055	Noise	Ischemic heart disease.
Ischemic heart disease	15-79	DALY	48552	Pb	Ischemic heart disease.
Cerebrovascular disease	15-79	DALY	29263	Pb	Cerebrovascular disease.
Hypertensive heart disease	15-79	DALY	3996	Pb	Hypertensive heart disease.
Other cardiac diseases	15-79	DALY	13773	Pb	Cardiomyopathy and myocarditis; atrial fibrillation and flutter; aortic aneurysm, peripheral vascular disease; endocarditis, other cardiovascular and circulatory diseases

**Table 3:** DALYs due to local transport in the study area.

**Factor group**	**Factor and health effect**	**DALYs**	**%**
Air pollution	PM2.5: natural mortality	16,994	67
	PM2.5: chronic bronchitis (COPD)	577	2
	PM2.5: restricted activity days (RAD)	138	1
	PM2.5: LRS symptoms days (school children)	35	0
	PM2.5: LRS symptoms days (adult)	80	0
	PM2.5–10: LRS symptoms days (school children)	107	0
	PM2.5–10: LRS symptoms days (adult)	246	1
	PM2.5–10: chronic bronchitis (COPD)	1774	7
	NO_*x*_: natural mortality	5135	20
	SO_2_: lung cancer	32	0
	BaP: lung cancer	0.02	0
	Cd: cancer	0.04	0
	Ni: cancer	0.003	0
	Pb: mild mental retardation (children)	8	0
	Pb: cardiovascular diseases (adult)	184	1
Air pollution	Air pollution together	25310	100
Noise	Cardiovascular disease	3394	13
	Sleep disturbance	9993	38
	Annoyance	13111	49
Noise	Noise total	26498	100
Traffic injuries	Injuries and fatalities	6276	100
Physical activity	Physical activity	−17309	100
Total	Together	40775	–
