# Supplementary material for: Burden of disease caused by local transport in Warsaw, Poland
Source: J Transp Health. 2015 Sep;2(3):423–33. doi: 10.1016/j.jth.2015.06.005 (PMC4557416; doi:10.1016/j.jth.2015.06.005)
Supplement: Supplementary file 1 — Supplementary material [file mmc1.docx]

**Burden of disease caused by local transport in Warsaw, Poland: Supplementary material**

Marko Tainio^1,2^

1. UKCRC Centre for Diet and Activity Research (CEDAR), MRC Epidemiology Unit, University of Cambridge School of Clinical Medicine, Box 285 Institute of Metabolic Science, Cambridge Biomedical Campus, Cambridge, CB2 0QQ, United Kingdom

2. Systems Research Institute, Polish Academy of Sciences, Newelska 6, 01-447 Warsaw, Poland.

1. **General information**

**Table S1**: Key data sources.

| **Data** | **Details** | **Year** | **Reference** |
| --- | --- | --- | --- |
| Global Burden of Disease | Background years of life lost due to premature mortality or fatality (YLL), disability-adjusted life-years (DALY) and mortality for Poland. | 2010 | (Global Burden of Disease Study 2010, 2013) |
| Air pollution, exposure | Air pollution concentration caused by local transport (see Table S3 for details). | 2005 | (Holnicki and Nahorski, 2013; Tainio et al., 2014) |
| Blood level Pb concentration in children | Measured Pb levels in blood, hair and teeth from 300 preschool age children in Southern Poland. | 1997–2004 | (Barton, 2011) |
| Blood level Pb concentration in adults | Blood lead levels in adults. | Undefined | (Fewtrell et al., 2003) |
| Traffic fatalities | Number of traffic fatalities. | 2009 | (Capital City of Warsaw, 2010) and personal information from Police |
| Noise | Day, evening and night (L_den_) and night (L_night_) (23:00-7:00) noise levels. | Undefined | (European Environment Agency (EEA), n.d.) |
| Walking and cycling levels | Year 2005 Warsaw traffic survey. | 2005 | (Capital City of Warsaw, 2005) |

**Table S2**: Disability weight and duration data.

| **Disease** | **Disability weight** | **Reference** | **Average duration (years)** | **Reference** |
| --- | --- | --- | --- | --- |
| Chronic bronchitis  (new cases) | 0.05 | Based on Hofstetter 1998 (Hofstetter, 1998) | 40 | Based on Hofstetter 1998 (Hofstetter, 1998) |
| Restricted activity days (RAD) | 0.099 | Based on EBoDE (Hänninen and Knol, 2011) | 0.00274 | Based on EboDe (Hänninen and Knol, 2011) |
| LRS symptoms days (school children) | 0.279 (0.279-0.280) | Lower respiratory infections (chronic sequelae) (World Health Organization, n.d.) | 0.00274 | One day. Based on EboDe (Hänninen and Knol, 2011) |
| LRS symptom days (adult) | 0.279 (0.279-0.280) | Lower respiratory infections (chronic sequelae) 23) | 0.00274 | One day. Based on EboDe (Hänninen and Knol, 2011) |
| Mild Mental Retardation (MMR) for children | 0.36 | EboDE (Hänninen and Knol, 2011) | 77.6 | EboDE (Hänninen and Knol, 2011) |
| Sleep disturbance | 0.07 (0.04-0.10) | (World Health Organization, 2011) | 1 | (World Health Organization, 2011) |
| High annoyance | 0.02 (0.01-0.12) | (World Health Organization, 2011) | 1 | (World Health Organization, 2011) |

1. **Health effects of air pollution**

**Table S3**: Mean exposure to transport generated air pollution in Warsaw, Poland (Holnicki and Nahorski, 2013; Tainio et al., 2014).

| **Pollutant** | **Mean exposure (µg/m^3^)** |
| --- | --- |
| PM_2.5_ | 4.7 |
| PM_2.5-10_ | 14 |
| SO_2_ | 1.5 |
| NO_x_ | 20 |
| BaP | 0.00000061 |
| Cd | 0.0000027 |
| Ni | 0.000027 |
| Pb | 0.010 |

- 1. **Particulate matter (PM_2.5_, PM_2.5-10_)**

*Natural-cause mortality*. The YLLs due to PM_2.5_ were estimated with the following equation:

PAF= (HR^(1/Eb))^E (S1)

YLL = PAF x YLL_Natural-cause mortality_ (S2)

Where PAF is the population attributable fraction, HR is the hazard rate for natural-cause mortality per 5 µg/m^3^ change in PM_2.5_ exposure, E is the exposure for PM_2.5_ (unit µg/m^3^), Eb is the PM_2.5_ exposure increment to which the HR is related (5 µg/m^3^), YLL_Natural-couse mortality_ is the background YLLs due to natural-cause mortality, and the YLL is the disease burden caused by PM_2.5_ related mortality.

For the HR a value of 1.07 (95% confidence interval (CI) 1.02-1.13) was used, based on the multicenter *European Study of Cohorts for Air Pollution Effects* study (Beelen et al., 2013).

The background YLL data included YLLs caused by the communicable, maternal, neonatal, and nutritional disorders, and non-communicable diseases (Table 2, Article). In (Beelen et al., 2013) the natural-cause mortality was defined to be deaths due to International Classification of Diseases (ICD)-9 codes 001–779 and ICD-10 codes A00–R99. Causes of deaths not directly associated to air pollution, such as injury, injuries and suicides, were excluded. Therefore injuries were excluded from background YLLs. Most of the cohort studies included in the (Beelen et al., 2013) were for adult populations and therefore PM_2.5_ was assumed to increase natural-cause mortality for the age group 30, or older.

*New cases of chronic bronchitis, RADs, LRS symptoms days for school children and LRS symptoms days for adults*. The YLDs for morbidity outcomes were estimated with equations:

AI_k_ = E_k_ x UR_k_ (S3)

DALY_k_=AI_k_ x DW_k_ x D_k_ (S4)

Where UR_k_ is the unit risk for disease k, AI_k_ is the Attributable Incidence (number of new cases per year) for disease k, E is the exposure level (PM_2.5_ or PM_2.5-10_), DW_k_ is the Disability Weight for disease k, and D_k_ is the duration of condition in years for disease k.

The unit risk values for different morbidity outcomes were adopted from the *Clean Air for Europe* (CAFE) program report (Hurley et al., 2005). The URs are summarized in Table 1 (Article) and the DWs and Ds in the Table S2 (Supplementary material). The UR uncertainty was quantified with triangular distributions and based on the values from the CAFE report (Table 1, Article). For the LRS days for adults, 30% of the adult population was estimated to have chronic respiratory symptoms, with uncertainty range from 20% to 50% (Hurley et al., 2005).

- 1. **Nitrogen oxides (NO_x_)**

The YLLs due to exposure to NO_x_ was estimated with equations S1 and S2 by using the HR value of 1.02 (95% CI 1.00-1.04) per 20 µg/m^3^ change in NO_x_ concentration. The HR was based on same (Beelen et al., 2013) cohort study as the HR for the PM_2.5_ (Table 1, Article).

- 1. **Sulfur dioxide (SO_2_)**

The DALYs due to SO_2_ related lung cancers were estimated with following equations:

RR = exp(E x ln(RR)/Eb) (S5)

PAF = (RR – 1) / RR (S6)

DALY = PAF x DALY_Lung cancer_ (S7)

Where RR is relative risk per 10 µg/m^3^ changes in SO_2_ exposure, E is the exposure for SO_2_, Eb is the exposure increment to which the RR is related (10 µg/m^3^), PAF is population attributable fraction and DALY_Lung cancer_ is the background lung cancer DALY in the study area. The RR was adopted from (Nafstad et al., 2003) study that followed a cohort of 16 209 Norwegian men with 27 year follow up time. The resulting RR associated to 10 µg/m^3^ change in SO_2_ concentration was 1.01 (95% CI 0.94-1.08) (Nafstad et al., 2003).

- 1. **Benzo(a)pyrene (BaP)**

The DALYs due to lung cancer caused by BaP was calculated with following equations:

AI = E x UR x Pop (S8)

DALY = (AI/75)*DALY_b_/death_b_ (S9)

Where E is exposure to BaP (unit ng/m^3^), UR is a life time cancer risk of lung cancer, Pop is the size of the study population and AI is the Attributable Incidence (number of new cases per year). The life time cancer risk of the population was divided with the 75 years to estimate new cases of cancers per year by assuming the average life span of 75 years. The number of new cancer cases per year was converted to DALYs by multiplying the number of cases with the mean DALY loss of one lung cancer (21.4 DALYs per lung cancer death), estimated from the GBD 2010 data for Poland (Global Burden of Disease Study 2010, 2013).

For the UR a value of 8.7 x 10^-5^ cancers per ng/m^3^ exposure to BaP was used, based on the WHO Air Quality guidelines for Europe (World Health Organization, 2000). The upper and lower bound values of 10 x 10^-5^ per ng/m^3^ and 1.0 x 10^-5^ per ng/m^3^, respectively, were used based on the summary of risk estimates for BaP from (Bostrom et al., 2002) review.

- 1. **Cadmium (Cd)**

The DALYs due to lung cancer caused by lifetime exposure to Cd were estimated with UR approach by adopting the equations S8 and S9. For UR a value of 1.8 x 10^-3^ cancers per µg/m^3^ was used. The same unit risk value was used in the ExternE year 2005 update (Bickel and Friedrich, 2005). For upper and lower band values of 9.2 x 10^-2^ and 1.0 x 10^-3^ were used, based on the (Takenaka et al., 1983) and author judgment, respectively.

- 1. **Nickel (Ni)**

The DALYs due to lung cancer cases caused by lifetime exposure to Ni were estimated with UR approach by adopting the equations S8 and S9. For UR a value of 2.4 x 10^-4^ cancers per µg/m^3^ was used. The same UR was used in ExternE (Bickel and Friedrich, 2005) and it is based on the inhalation UR value from United States Environmental Protection Agency (US EPA) Integrated Risk Information System (IRIS) database (United States Environmental Protection Agency, n.d.). For upper and lower bounds values of 4.6 x 10^-4^ and 1.1 x 10^-5^ were used, based on (Peto et al., 1984) and (Chovil et al., 1981), respectively.

- 1. **Lead (Pb)**

The adverse health effects of Pb exposure were estimated by following the WHO burden of disease guidelines for lead (Fewtrell et al., 2003). Due to the non-linear nature of the dose-response relationship between Pb and the associated health effects, the total health burden due to Pb in the study area was first calculated and then the fraction of that burden due to local traffic related Pb emissions was estimated.

The average Pb concentrations in the air were converted to blood Pb levels by assuming that 1.0 µg/m^3^ increase of Pb in the air leads to 50 μg/L increase in blood level Pb levels, following a similar approach taken in the ExternE (Bickel and Friedrich, 2005). For the sensitivity analysis +/-25% uncertainty around this conversation factor was estimated. The background blood Pb levels for children and adults were estimated based on the local exposure study and to WHO recommended values, respectively (see below for details).

*Mild Mental Retardation (MMR)*. The exposure to Pb in early childhood has been associated with the decreased intelligence (Lanphear et al., 2005). For some individuals the decrease in the intelligence quotient (IQ) due to Pb leads to MMR. In (Fewtrell et al., 2003) mental retardation was estimated to be mild when IQ drops below 70 points.

**Table S4**: Calculation of Mild Mental Retardation (MMR) due to Pb. The calculations follows the methods presented in the (Fewtrell et al., 2003).

| Blood lead level intervals | Proportion of children at risk (H) | Fraction of population in IQ interval (I) | Fraction of population at risk with given exposure (H x I) | Adjustment factor for EUR-B area | Number of 0-1 year old children in Warsaw, Poland | Number of new cases of MMRs per year  (J x K x L) |
| --- | --- | --- | --- | --- | --- | --- |
| <5 µg/dl | 0.737 | - | - | - | - | - |
| 5-10 µg/dl | 0.253 | 0.24 | 0.00061 | - | - | - |
| 10-15 µg/dl | 0.01 | 0.8 | 0.00008 | - | - | - |
| 15-20 µg/dl | 0 | 1.45 | 0 | - | - | - |
| 20> µg/dl | 0 | 1.59 | 0 | - | - | - |
| **Total** | **-** |  | **0.00069** | **1.53** | **18566** | **19.52** |

The background blood level Pb concentrations in children were estimated from the Barton (2011). In that study the Pb and Cd levels in blood, hair and teeth were measured for 300 preschool age children in Southern Poland. The geometric mean blood level Pb concentrations were 42 µg/l and geometric standard deviation 1.5 for children living in urban area (n=99). This is close to the regional blood level of 58 µg/l for children in Poland, Turkey and Yugoslavia, presented in the appendix of the (Fewtrell et al., 2003).

By assuming the blood level geometric mean concentration of 42 µg/l and variation with the geometric standard deviation of 1.5, the children were divided into five different exposure groups based on the lead levels in their blood (Table S4, Supplementary material). The proportion of children at risk in each exposure group was then multiplied with the fraction of population in each IQ interval (based on Table 2 in Fewtrell et al. (2003) and the review by Schwartz (1994)). The IQ intervals represent the fraction of population that could potentially develop MMR if their IQ would decrease as a result of Pb exposure. For example, with the exposure group of 5-10 µg/dl, the background IQ interval in risk is in between 70.00 and 70.65 IQ points, and these children would develop MMR if their IQ would drop due to Pb more than 0.65 IQ points.

The fraction of the population at risk of developing MMR in the study area was calculated by first summing at risk population in each exposure intervals and then multiplying the fraction with the regional adjustment factors. Regional adjustment factors are used to take into account that several other stressors are causing MMR and by using the adjustment factor the combined effect of Pb and these other stressors can be estimated. For Poland the adjustment factor is 1.53 (EurB-group in Table 3, (Fewtrell et al., 2003)). The number of new MMR cases per year is then calculated by multiplying the adjusted at-risk population with the number of 0-1 year old children in the study area. To calculate the DALYs, the number of new MMR cases per year was multiplied with the disability weight of 0.36 and with the average duration of 77.6 years (Table S2, Supplementary material).

By converting the annual average Pb concentration in the air to blood level concentration by using the conversation factor from ExternE (Bickel and Friedrich, 2005) study (see details earlier), 1.4% of total burden of Pb for children was estimated to be due to air pollution emissions from local transport.

*Cardiovascular disease*. The calculation of new cases of cardiovascular diseases due to Pb followed similar pattern as the calculation of MMRs for children. For the background blood level concentrations, the regional blood level of 9.2 µg/dl was used with the standard deviation of 3 for adults in Poland, Turkey and Yugoslavia, as presented in the appendix of the Fewtrell et al. (2003). Based on this background exposure, population was divided into five exposure groups using the same method as was used in the MMR calculations (Table S4, Supplementary material).

**Table S5**: Relative risk values for cardiovascular disease for different blood level Pb’s. Adopted from (Fewtrell et al., 2003) and based on (Pruss-Ustun et al., 2004).

| **Gender, disease** | **Age group** | | | | |
| --- | --- | --- | --- | --- | --- |
| **Male, <5** µ**g/dl** | **15–29** | **30–44** | **45–59** | **60–69** | **70–79** |
| Ischaemic heart disease | 1.000 | 1.000 | 1.000 | 1.000 | 1.000 |
| Cerebrovascular disease | 1.000 | 1.000 | 1.000 | 1.000 | 1.000 |
| Hypertensive disease | 1.000 | 1.000 | 1.000 | 1.000 | 1.000 |
| Other cardiac diseases | 1.000 | 1.000 | 1.000 | 1.000 | 1.000 |
| **Male, 5-10** µ**g/dl** |  |  |  |  |  |
| Ischaemic heart disease | 1.041 | 1.041 | 1.032 | 1.018 | 1.014 |
| Cerebrovascular disease | 1.056 | 1.056 | 1.044 | 1.029 | 1.020 |
| Hypertensive disease | 1.122 | 1.122 | 1.059 | 1.036 | 1.027 |
| Other cardiac diseases | 1.013 | 1.013 | 1.009 | 1.006 | 1.003 |
| **Male, 10-15** µ**g/dl** |  |  |  |  |  |
| Ischaemic heart disease | 1.130 | 1.130 | 1.100 | 1.055 | 1.043 |
| Cerebrovascular disease | 1.177 | 1.177 | 1.137 | 1.089 | 1.061 |
| Hypertensive disease | 1.413 | 1.413 | 1.189 | 1.111 | 1.083 |
| Other cardiac diseases | 1.039 | 1.039 | 1.026 | 1.017 | 1.010 |
| **Male, 15-20** µ**g/dl** |  |  |  |  |  |
| Ischaemic heart disease | 1.225 | 1.225 | 1.172 | 1.093 | 1.072 |
| Cerebrovascular disease | 1.312 | 1.312 | 1.239 | 1.152 | 1.104 |
| Hypertensive disease | 1.779 | 1.779 | 1.334 | 1.192 | 1.142 |
| Other cardiac diseases | 1.067 | 1.067 | 1.044 | 1.029 | 1.017 |
| **Male, 20>** µ**g/dl** |  |  |  |  |  |
| Ischaemic heart disease | 1.276 | 1.276 | 1.210 | 1.112 | 1.087 |
| Cerebrovascular disease | 1.385 | 1.385 | 1.293 | 1.185 | 1.126 |
| Hypertensive disease | 1.996 | 1.996 | 1.413 | 1.235 | 1.172 |
| Other cardiac diseases | 1.081 | 1.081 | 1.053 | 1.035 | 1.02 |
| **Female <5** µ**g/dl** | **15–29** | **30–44** | **45–59** | **60–69** | **70–79** |
| Ischaemic heart disease | 1.000 | 1.000 | 1.000 | 1.000 | 1.000 |
| Cerebrovascular disease | 1.000 | 1.000 | 1.000 | 1.000 | 1.000 |
| Hypertensive disease | 1.000 | 1.000 | 1.000 | 1.000 | 1.000 |
| Other cardiac diseases | 1.000 | 1.000 | 1.000 | 1.000 | 1.000 |
| **Female, 5-10** µ**g/dl** |  |  |  |  |  |
| Ischaemic heart disease | 1.026 | 1.026 | 1.021 | 1.011 | 1.009 |
| Cerebrovascular disease | 1.035 | 1.035 | 1.028 | 1.018 | 1.013 |
| Hypertensive disease | 1.076 | 1.076 | 1.038 | 1.023 | 1.017 |
| Other cardiac diseases | 1.008 | 1.008 | 1.005 | 1.004 | 1.002 |
| **Female, 10-15** µ**g/dl** |  |  |  |  |  |
| Ischaemic heart disease | 1.081 | 1.081 | 1.063 | 1.035 | 1.027 |
| Cerebrovascular disease | 1.11 | 1.11 | 1.086 | 1.056 | 1.039 |
| Hypertensive disease | 1.247 | 1.247 | 1.117 | 1.07 | 1.052 |
| Other cardiac diseases | 1.025 | 1.025 | 1.017 | 1.011 | 1.006 |
| **Female, 15-20** µ**g/dl** |  |  |  |  |  |
| Ischaemic heart disease | 1.139 | 1.139 | 1.107 | 1.058 | 1.046 |
| Cerebrovascular disease | 1.19 | 1.19 | 1.147 | 1.095 | 1.065 |
| Hypertensive disease | 1.446 | 1.446 | 1.203 | 1.119 | 1.088 |
| Other cardiac diseases | 1.042 | 1.042 | 1.028 | 1.018 | 1.011 |
| **Female, 20>** µ**g/dl** |  |  |  |  |  |
| Ischaemic heart disease | 1.169 | 1.169 | 1.13 | 1.07 | 1.055 |
| Cerebrovascular disease | 1.232 | 1.232 | 1.179 | 1.115 | 1.079 |
| Hypertensive disease | 1.556 | 1.556 | 1.248 | 1.145 | 1.107 |
| Other cardiac diseases | 1.051 | 1.051 | 1.033 | 1.022 | 1.013 |

Increased systolic blood pressure is associated with an increase in four different diseases: ischemic heart disease, cerebrovascular disease, hypertensive disease and other cardiac diseases. The RRs for each disease, exposure interval, age group and gender were obtained from Fewtrell et al. (2003) (Table S5, Supplementary material). By combining the exposure data with the RRs DALYs were calculated with the following equations:

PAF_j,k_ = (∑_l_(P_l_xRR_l,j,k_)-1)/ ∑_l_(P_l_RR_l_ ) (S10)

DALY = DALY_k_ x ∑_j_(PAF_j,k_) (S11)

Where P_l_ is the fraction of population in at exposure interval l, RR_l,j,k_ is the relative risk at exposure interval l, gender j and disease k, and DALY_k_ is the background DALY for disease k. By converting the annual average Pb concentration in the air to blood level concentration by using the conversation factor from the ExternE (Bickel and Friedrich, 2005) study (see details earlier), 6.1% of the total burden of Pb for adults was estimated to be due to air pollution emissions from local transport.

1. **Health effects of noise**

**Table S6**: Fraction of population exposed to different environmental noise levels in Warsaw. The noise data is from Eionet forum (European Environment Agency (EEA), n.d.) and ischemic heart disease and HSD data from (World Health Organization, 2011).

| Exposure group (dB(A)) | Fraction of population exposed to L_den_ | OR for ischemic heart diseases | Fraction of population exposed to L_night_ | Percentage of people highly sleep disturbed (HSD) (%) |
| --- | --- | --- | --- | --- |
| 45-49 | - | - | 0.24 | 4.453 |
| 50-54 | - | - | 0.24 | 6.633 |
| 55-59 | 0.16 | 1.00 | 0.24 | 9.556 |
| 60-64 | 0.24 | 1.015 | 0.16 | 13.22 |
| 65-69 | 0.24 | 1.067 | 0.07 | 17.63 |
| 70-74 | 0.15 | 1.161 | 0 | 20.11 |
| >75 | 0.04 | 1.302 | - | - |

- 1. **Noise: Ischemic heart diseases**

The DALYs due to noise related ischemic heart diseases were modelled based on the day, evening and night noise (L_den_) exposure levels. The DALYs were calculated with the equations:

PAF = (∑_l_(P_l_OR_l_)-1)/ ∑_l_(P_l_OR_l_) (S12)

DALY = DALY_ischemic heart diseases_ x PAF (S13)

Where OR_l_ is the odds ratio for the exposure interval l and P_l_ is the fraction of population in exposure interval l. Both P_l_ and OR_l_ values are presented in Table S6 (Supplementary material). The ORs were adapted from the Appendix 1 of the *Burden of disease from environmental noise* guide (World Health Organization, 2011).

The ORs for different L_den_ levels were based on polynomial fit of the association between road traffic noise and incidence of myocardial infarction (World Health Organization, 2011). As the *Burden of disease from environmental noise* discussed, the exposure-response relationship between myocardial infarction can be extended for all ischemic heart diseases for the calculation of the DALYs. The same approach was used in EBoDE study (Hänninen and Knol, 2011) and in the present study.

- 1. **Noise: High sleep disturbance (HSD)**

The percentage of people with HSD due to road traffic noise was calculated with following equation:

HSD(%) = 20.8 – 1.05(L_night_)+0.01486(L_night_)^2 (S14)

Where L_night_ is the average night noise level and HSD(%) the percentage of population with HSD. The polynomial function presented in equation 14 was developed in the (Miedema et al., 2003) based on self-reported sleep disturbance from 15 different data sets. The polynomial function was approximated based on data from noise range 45-65 dB(A). In the *Burden of disease from environmental noise* guidelines the function was approximated also for higher exposure levels (>65 dB(A)) (World Health Organization, 2011). The same approach was used in this study.

The L_night_ exposure levels and the percentage of people with HSD, based on the equation 14, are presented in Table S6 (Supplementary material). The percentage of the population exposed for 45-49 dB(A) was estimated to be same as the percentage of the population exposed to 50-54 dB(A), following similar approach used in (World Health Organization, 2011). The DALYs due to sleep disturbance were calculated by multiplying the number of people with HSD with the median disability weight of 0.07 with an uncertainty range of 0.04-0.10,and a duration of one year (Table S2, Supplementary material) (World Health Organization, 2011).

- 1. **Noise: Annoyance**

**Table S7**: Calculation of the fraction of population annoyed. The percentage of population annoyed in each noise intervals has been calculated by applying the equation S15.

|  | Fraction of population exposed to L_den_ | Percentage of population annoyed |
| --- | --- | --- |
| <55 | 0.17 | 2.8 |
| 55-59 | 0.16 | 8.1 |
| 60-64 | 0.24 | 13 |
| 65-69 | 0.24 | 20 |
| 70-74 | 0.15 | 30 |
| >75 | 0.04 | 30 |

The number of people annoyed due to noise was calculated with similar methods to the number of people developing HSD due to noise. First, the percentage of the population annoyed due to noise in different noise exposure groups was estimated with following equation:

((((7.239*(10^-4))*((L_den_-42)^3))-((7.851*(10^-3))*(( L_den_ -42)^2)))+(0.1695*( L_den_ -42))) (S15)

Where L_den_ is the day, evening, night exposure level for five dB(A) intervals (Table S6, Supplementary material). The equation S15 was obtained from the Miedema and Oudshoorn (2001) study that estimated the functional relationship between noise exposure and the percentage of the population annoyed. The DALYs due to annoyance were calculated by multiplying the number of people annoyed with the disability weight of 0.02 (median) with an uncertainty range from 0.01 to 0.12 and with the average duration of one year (Table S2, Supplementary material).

1. **Health effects of physical activity**

**Table S8**: Percentage of trips made by walking, cycling and public transport, the average duration of the trips and the METs generated while doing the activity. Trips per mode and average duration based on (Capital City of Warsaw, 2005) and METs to (Ainsworth et al., 2011).

| Mode of transport | % of trips with the mode | | The average duration of the trip (min) | | Median METs and uncertainty range |
| --- | --- | --- | --- | --- | --- |
| - | Week day | Saturday | Week day | Saturday | - |
| Walking | 21% | 19.4% | 17 | 18 | 3.5 (2.0 - 5-0) |
| Cycling | 0.9% | 1.7% | 24 | 46 | 6.8 (4.0 - 8.0) |
| Public transport | 54.2% | 45.2% | 5 (1-10) | 5 (1-10) | 2.5 (2.0 - 3.0) |

Note: For public transport average duration and METs refers to walking to and from to public transport.

The YLLs due to physical activity were estimated with the following equations:

PAF= (RR^(1/Eb))^MET (S16)

YLL = PAF x YLL_All-cause mortality_ (S17)

Where PAF is the population attributable fraction, RR is the relative risk for all-cause mortality per 11.25 METh/week change in walking or cycling, MET is the exposure for walking and cycling (unit METh/week), Eb is the exposure increment to which the RR is related (11.25 METh/week), YLL_All-cause mortality_ is the background YLLs due to all-cause mortality, and the YLL is the disease burden caused by physical activity. RRs for walking and cycling were obtained from Kelly et al (2014) (log-linear RRs in Table 2 of that article). The average exposure to active transport related physical activity is described in article manuscript and in Table S8.

1. **Results**

**Table S9:** Comparison of the result to (Kjellstrom et al., 2008).

|  | Kjellström et al. 2008 | | Present study | |
| --- | --- | --- | --- | --- |
| Factor | DALY | DALY/million inhabitants | DALY | DALY/million inhabitants |
| Injury | 25,000 | 2,810 | 6,276 | 3,692 |
| Air pollution | 35,000 | 3,934 | 25,310 | 14,888 |
| Noise | 4,000 | 450 | 26,498 | 15,587 |
| Physical inactivity | 38,000 | 4,272 | -17,309 | -10,182 |

**References**

Ainsworth, B.E., Haskell, W.L., Herrmann, S.D., Meckes, N., Bassett, D.R.J., Tudor-Locke, C., Greer, J.L., Vezina, J., Whitt-Glover, M.C., Leon, A.S., 2011. 2011 Compendium of Physical Activities: a second update of codes and MET values. Med Sci Sports Exerc 43, 1575–1581. doi:10.1249/MSS.0b013e31821ece12

Barton, H.J., 2011. Advantages of the Use of Deciduous Teeth, Hair, and Blood Analysis for Lead and Cadmium Bio-Monitoring in Children. A Study of 6-Year-Old Children from Krakow (Poland). Biol. Trace Elem. Res. 143, 637–658. doi:10.1007/s12011-010-8896-6

Beelen, R., Raaschou-Nielsen, O., Stafoggia, M., Andersen, Z.J., Weinmayr, G., Hoffmann, B., Wolf, K., Samoli, E., Fischer, P., Nieuwenhuijsen, M., Vineis, P., Xun, W.W., Katsouyanni, K., Dimakopoulou, K., Oudin, A., Forsberg, B., Modig, L., Havulinna, A.S., Lanki, T., Turunen, A., Oftedal, B., Nystad, W., Nafstad, P., De Faire, U., Pedersen, N.L., Östenson, C.-G., Fratiglioni, L., Penell, J., Korek, M., Pershagen, G., Eriksen, K.T., Overvad, K., Ellermann, T., Eeftens, M., Peeters, P.H., Meliefste, K., Wang, M., Bueno-de-Mesquita, B., Sugiri, D., Krämer, U., Heinrich, J., de Hoogh, K., Key, T., Peters, A., Hampel, R., Concin, H., Nagel, G., Ineichen, A., Schaffner, E., Probst-Hensch, N., Künzli, N., Schindler, C., Schikowski, T., Adam, M., Phuleria, H., Vilier, A., Clavel-Chapelon, F., Declercq, C., Grioni, S., Krogh, V., Tsai, M.-Y., Ricceri, F., Sacerdote, C., Galassi, C., Migliore, E., Ranzi, A., Cesaroni, G., Badaloni, C., Forastiere, F., Tamayo, I., Amiano, P., Dorronsoro, M., Katsoulis, M., Trichopoulou, A., Brunekreef, B., Hoek, G., 2013. Effects of long-term exposure to air pollution on natural-cause mortality: an analysis of 22 European cohorts within the multicentre ESCAPE project. The Lancet.

Bickel, P., Friedrich, R., 2005. ExternE - Externalities of Energy - Methodology 2005 Update (EUR 21951). European Commission.

Bostrom, C.E., Gerde, P., Hanberg, A., Jernstrom, B., Johansson, C., Kyrklund, T., Rannug, A., Tornqvist, M., Victorin, K., Westerholm, R., 2002. Cancer risk assessment, indicators, and guidelines for polycyclic aromatic hydrocarbons in the ambient air. Environ. Health Perspect. 110, 451–488.

Capital City of Warsaw, 2005. WARSZAWSKIE BADANIE RUCHU 2005 WRAZ Z OPRACOWANIEM MODELU RUCHU. Capital City of Warsaw, Warsaw, Poland.

Chovil, A., Sutherland, R., Halliday, M., 1981. Respiratory cancer in a cohort of nickel sinter plant workers. British Journal of Industrial Medicine 38, 327–333.

European Environment Agency (EEA), n.d. The European environment information and observation network (EIONET) Noise database [WWW Document]. Eionet forum. URL http://forum.eionet.europa.eu/etc-sia-consortium/library/noise_database/index_html (accessed 5.21.13).

Fewtrell, L., Kaufmann, R., Pruss-Ustun, A., 2003. Lead assessing the environmental burden of disease at national and local levels. World Health Organization, [Geneva].

Global Burden of Disease Study 2010, 2013. Poland Global Burden of Disease Study 2010 (GBD 2010) Results 1990-2010. Institute for Health Metrics and Evaluation (IHME), Seattle, United States.

Hänninen, O., Knol, A., 2011. European Perspectives on Environmental Burden of Disease Estimates for Nine Stressors in Six European Countries (No. 1/2011). National Institute for Health and Welfare (THL), Finland, Helsinki, Finland.

Hofstetter, P., 1998. Perspectives in life cycle impact assessment: a structured approach to combine models of the technosphere, ecosphere, and valuesphere. Kluwer Academic, Boston.

Holnicki, P., Nahorski, Z., 2013. Air quality modeling in the Warsaw Metropolitan Area. Journal of Theoretical and Applied Computer Science 7, 56–69.

Hurley, F., Hunt, A., Cowie, H., Holland, M., Miller, B., Pye, S., Watkiss, P., 2005. Methodology Paper (Volume 2) for Service Contract for carrying out cost-benefit analysis of air quality related issues, in particular in the clean air for Europe (CAFE) programme (No. AEAT/ED51014/Methodology Volume 2: Issue 2). AEA Technology Environment, United Kingdom.

Kelly, P., Kahlmeier, S., Goetschi, T., Orsini, N., Richards, J., Roberts, N., Scarborough, P., Foster, C., 2014. Systematic review and meta-analysis of reduction in all-cause mortality from walking and cycling and shape of dose response relationship. Int. J. Behav. Nutr. Phys. Act. 11, 132. doi:10.1186/s12966-014-0132-x

Kjellstrom, T., Ferguson, R., Taylor, A., 2008. Health Impact Assessment of road transport in Sweden: A discussion paper describing the development and testing of HIA methodology. Report from a research project for the Swedish Road Administration, 2007 (No. 2009:67), Health impact assessment and public health costs of the road transport sector – Results from two projects. Swedish Road Administration, Borlänge, Sweden.

Lanphear, B.P., Hornung, R., Khoury, J., Yolton, K., Baghurst, P., Bellinger, D.C., Canfield, R.L., Dietrich, K.N., Bornschein, R., Greene, T., Rothenberg, S.J., Needleman, H.L., Schnaas, L., Wasserman, G., Graziano, J., Roberts, R., 2005. Low-level environmental lead exposure and children’s intellectual function: an international pooled analysis. Environ Health Perspect 113, 894–899.

Miedema, H.M., Oudshoorn, C.G., 2001. Annoyance from transportation noise: relationships with exposure metrics DNL and DENL and their confidence intervals. Environ Health Perspect 109, 409–416.

Miedema, H.M.., Passchier-Vermeer, Vos, 2003. Elements for a position paper on night-time transportation noise and sleep disturbance. TNO Inro, Delft.

Nafstad, P., Haheim, L.L., Oftedal, B., Gram, F., Holme, I., Hjermann, I., Leren, P., 2003. Lung cancer and air pollution: a 27 year follow up of 16 209 Norwegian men. Thorax 58, 1071–1076. doi:10.1136/thorax.58.12.1071

Peto, J., Cuckle, H., Doll, R., Hermon, C., Morgan, L., 1984. Respiratory cancer mortality of Welsh nickel refinery workers. IARC Sci. Publ. 37–46.

Pruss-Ustun, A., Fewtrell, L., Landrigan, P.J., Ayuso-Mateos, J.., 2004. Lead exposure, in: Comparative Quantification of Health Risks. Global and Regional Burden of Disease Attributable to Selected Major Risk Factors. Volume 2. World Health Organization, Geneva, Switzerland.

Schwartz, J., 1994. Low-level lead exposure and children’s IQ: a meta-analysis and search for a threshold. Environ Res 65. doi:10.1006/enrs.1994.1020

Tainio, M., Holnicki, P., Loh, M.M., Nahorski, Z., 2014. Intake Fraction Variability Between Air Pollution Emission Sources Inside an Urban Area. Risk Anal. doi:10.1111/risa.12221

Takenaka, S., Oldiges, H., Konig, H., Hochrainer, D., Oberdorster, G., 1983. CARCINOGENICITY OF CADMIUM CHLORIDE AEROSOLS IN W RATS. J. Natl. Cancer Inst. 70, 367–373.

United States Environmental Protection Agency, n.d. Integrated Risk Information System (IRIS) [WWW Document]. Integrated Risk Information System (IRIS). URL http://www.epa.gov/iris/

World Health Organization, 2011. Burden of disease from environmental noise: Quantification of healthy life years lost in Europe. World Health Organization (WHO), Copenhagen, Denmark.

World Health Organization, 2000. Air quality guidelines for Europe, 2nd ed. ed, WHO regional publications. World Health Organization, Regional Office for Europe, Copenhagen.

World Health Organization, n.d. GLOBAL BURDEN OF DISEASE 2004 UPDATE: DISABILITY WEIGHTS FOR DISEASES AND CONDITIONS.
